# Effects of the hangover secret on mitigating hangover symptoms: A pilot study

**DOI:** 10.1002/hsr2.330

**Published:** 2021-07-16

**Authors:** Der Yu Wang, Sheel Patel, Kimberly Maiton, Kevin Pham, Kate M. O'Dell, Nancy N. Nguyen, Sachin A. Shah

**Affiliations:** ^1^ Department of Pharmacy Practice Thomas J. Long School of Pharmacy and Health Sciences, University of the Pacific Stockton California USA; ^2^ Clinical Investigations Facility; David Grant USAF Medical Center Travis AFB California USA

**Keywords:** acute hangover scale, acute hangover severity scale, alcohol hangover, binge drinking, breath alcohol concentration, headache

## Abstract

**Background:**

Due to the popularity of excessive alcohol consumption, there is an increasing need for hangover symptom remedies. Most commercially available hangover treatment products have not been tested for efficacy through clinical study.

**Aims:**

The purpose of this pilot study was to characterize the activity of a commercially available hangover product, The Hangover Secret (THS).

**Methods:**

This was a randomized, double‐blinded, placebo‐controlled, crossover pilot study. Healthy volunteers of 21‐ to 40‐years‐old were eligible for participation, and received either THS or placebo on two different occasions. Participants were given 43 mL of whiskey every twenty minutes for up to 3 hours to achieve a blood alcohol concentration (BrAC) ≥ 0.12%. Hangover severity was assessed using the Acute Hangover Scale (AHS) and Acute Hangover Severity Scale (AHSS) validated tools.

**Results:**

Nine participants completed the study. AHS scores increased from baseline to 7 am by 4.11 ± 3.17 and 1.26 ± 2.29 for the placebo and active arms respectively (*P* = .16). AHS headache scores increased from baseline to 7 am by 2.44 ± 1.67 and 1.11 ± 1.17 for the placebo and active arms respectively (*P* = .06). AHSS scores increased from baseline to 7 am by 1.0 ± 1.05 and 0.41 ± 1.08, for the placebo and active arms respectively (*P* = .30). There was no significant difference between average BrAC at 7 am between the placebo and active arms.

**Conclusion:**

THS showed positive signals in the prevention of alcohol‐induced hangover, especially headaches. The improvements with THS surpassed the minimum clinically important difference in overall AHS score and three individual AHS symptoms scores (hangover, headache, and thirsty). THS's reduction in AHS or AHSS scores did not reach statistical significance likely due to the small sample size. Larger studies with appropriate sample sizes are needed in light of these promising findings.

## 1 INTRODUCTION

A hangover is defined as the combination of mental and physical symptoms experienced the day after a single episode of heavy drinking, starting when blood alcohol concentration approaches zero.^1^ Studies showed that 29% of undergraduate students reported losing school time to recover from hangovers^2^ and approximately 9% of U.S. laborers work with a hangover, which may adversely affect job performance.^3^ Most existing over‐the‐counter commercially available products do not have scientific data to support any claims.

The Hangover Secret (THS) is a powder packet mixed in water that contains milk thistle, N‐acetylcysteine, thiamine, pyridoxine, electrolytes, and an antioxidant superblend. Milk thistle, a main ingredient, has been shown in pre‐clinical and clinical data for its liver protective effect.^4^ The purpose of this pilot study was to assess the activity of THS in mitigating the symptoms of a hangover after a night of excessive alcohol consumption.

## 2 METHODS

This was a randomized, double‐blind, placebo‐controlled, cross‐over study. All participants were assigned to receive THS and a placebo (cherry syrup, lime, and water) with a minimum 6‐day washout period. A third party prepared THS and placebo to maintain blinding. Randomization was performed using a computer‐generated code. At each study visit, participants were to arrive at the study site at 6 pm to simulate a night of drinking. The first dose of THS or placebo was administered prior to the start of alcohol administration (8 pm) and a second dose given before sleep (12 am). Participants stayed over‐night and were discharged when Breath Alcohol Concentration (BrAC) reached 0 or when the investigator deemed it was safe to do so.

Healthy adults between the ages of 21 to 40 years old were eligible for inclusion and required to sign the Informed Consent Document. Participants who scored less than 5 on the Hangover Symptom Scale^5^ or denied experiencing a hangover before, were currently taking any prescription medications or supplements interacting with alcohol, or consumed caffeinated beverages consistently for more than 5 days per week were excluded. Participants who were pregnant, lactating, or anticipating pregnancy were also excluded.

The main endpoints were the change in scores for the Acute Hangover Scale (AHS), Alcohol Hangover Severity Scale (AHSS), and BrAC. AHS assesses the individual's symptoms with nine different factors; this test categorizes and ranks symptoms as 0 = none, 1 = mild, 4 = moderate, or 7 = incapacitating. AHSS is a validated, 12‐factor scoring system that ranks symptoms from 0 to 10 (0 = absence of symptom, 10 = extreme symptom).^6^ These categorical rankings were converted into numerical data based on a validated calculation.^7^ BrAC was defined as the breath alcohol concentration measured via breathalyzer.

On each of the study days, assessments of the AHS and AHSS were administered at baseline and upon subjects' awakenings the following day (Day 2) at 7 am. A standard breathalyzer (BACtrack S80) was used to determine the participants' BrAC prior to each administration of alcohol.

From 8 pm to 11 pm, participants were given 43 mL of Makers Mark whiskey (45% alcohol by volume), equivalent to 1 imperial shot of alcohol, every 20 minutes until either their BrAC was ≥0.12% or if the allotted time had been reached.^8^ Water consumption was recorded for 24‐hours prior to and throughout study days, and standardized meals were given on study visits.

All statistical analyses were performed using StatsDirect Version 3.1.18 (StatsDirect LTD). Wilcoxon signed‐rank test was used to compare overall AHS, AHSS, and BrAC scores.

## 3 RESULTS

**FIGURE 1:**
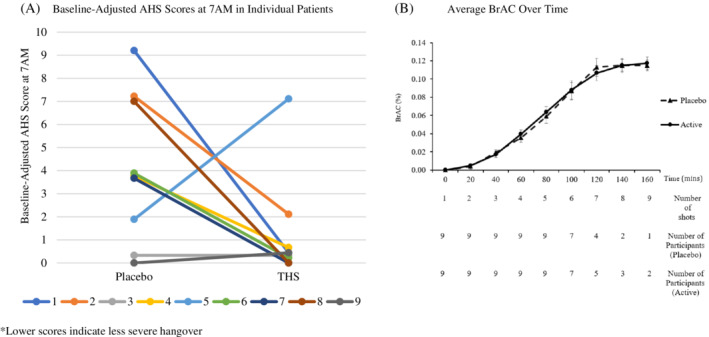
Change in AHS Scores and BrAC. (A) Individual patient level data show positive signals in AHS scores (downward trend). (B) Similar BrAC concentrations between placebo and The Hangover Secret arms

A total of 45 people were screened and 10 were enrolled into the study. One participant dropped out of the study due to a scheduling conflict. Data on nine participants were included for analyses (Figure 1A). The average age was 25 ± 2 years of age with 56% of participants being male and of Asian descent. The average BMI was 26.7 ± 2.80 kg/m^2^. The average systolic blood pressure and diastolic blood pressure among participants were 121 ± 9.77 mmHg and 72 ± 5.21 mmHg, respectively.

The average changes of AHS scores from baseline to 7 am were 4.11 ± 3.17 and 1.26 ± 2.29 for the placebo and active arms respectively (*P* = .16, Table 1). The AHS hangover scores changed by 2.22 ± 1.72 and 1.00 ± 1.22 from baseline to 7 am in the placebo and active arm respectively (*P* = .19). The AHS headache scores changed by 2.44 ± 1.67 and 1.11 ± 1.17 from baseline to 7 am in the placebo and active arms respectively (*P* = .06). The AHS thirsty score increased by 2.11 ± 2.03 and 0.89 ± 0.93 from baseline to 7 am in the placebo and active arm respectively (*P* = .20).

**TABLE 1:** Individual participants level data

Participants ID		1	2	3	4	5	6	7	8	9
Baseline AHS scores	Placebo	0.00	0.00	0.00	0.11	0.22	1.89	0.11	0.22	0.22
	Active	0.00	0.00	0.00	0.00	0.22	1.89	3.56	3.78	0.00
Change in AHS scores from baseline to 7 am	Placebo	9.22	7.22	0.33	3.78	1.89	3.89	3.67	7.00	0.00
	Active	0.44	2.11	0.33	0.67	7.11	0.22	0.00	0.00	0.44
Baseline AHSS scores	Placebo	0.00	0.17	0.00	0.00	2.33	0.83	0.33	0.67	0.33
	Active	0.00	0.00	0.00	0.00	1.33	1.42	0.83	2.58	0.00
Change in AHSS scores from baseline to 7 am	Placebo	3.08	1.58	0.00	1.50	−0.16	0.75	0.75	1.50	−0.08
	Active	0.42	1.67	0.25	0.75	2.25	−0.75	−0.50	−1.00	0.58
BrAC at 7 am (%)	Placebo	0.010	0.008	0.026	0.038	0.000	0.035	0.000	0.019	0.000
	Active	0.028	0.010	0.059	0.062	0.010	0.021	0.031	0.027	0.000
Number of shots	Placebo	6	6	7	8	5	7	6	9	5
	Active	6	6	8	9	5	7	7	9	5

*Note*: Individual data on baseline AHS score, change in AHS score at 7 am, baseline AHSS score, change in AHSS score at 7 am, BrAC were recorded, and amount of alcohol consumption are presented.

The average changes in AHSS scores from baseline to 7 am were 1.0 ± 1.05 and 0.41 ± 1.08, for the placebo and active arms respectively (*P* = .30).

The average BrAC and the total number of shots consumed for all participants over time in the placebo and active arms are shown in Figure 1B. All participants except for one exceeded a BrAC of 0.120% throughout both treatment arms. There were no statistically significant differences in BrAC between active and placebo arm throughout the administration of alcohol.

The average BrAC at 7 am in the placebo and active arms were 0.015% ± 0.015% and 0.028% ± 0.021%, respectively (*P* = .054). Three out of the nine participants consumed an additional shot of alcohol in the active arm compared to placebo. Additional participant level data is shown in Table 1.

Food consumption was approximately the same for both study days. Water consumption over a 36‐hour period was variable, with the smallest difference being 90 mL and the largest difference being 1990 mL (median = 684 mL).

## 4 DISCUSSION

In this randomized, double‐blinded, placebo‐controlled pilot study, THS's reduction in AHS or AHSS scores did not reach statistical significance due to small sample size. However, closer examination of individual patient‐level data revealed clinically relevant reductions in the overall AHS score and three individual AHS symptom scores, which warrants further investigation.

The minimum clinically important difference (MCID), calculated by applying the one‐half standard deviation criterion as a benchmark, is −0.31 for the overall AHS score and −0.84 for individual AHS symptom scores.^9^ THS improvements surpassed these MCIDs evident by the change in overall AHS score (−2.86) and the three individual AHS symptoms scores [hangover (−1.22), headache (−1.33), and thirsty (−1.22)]. In addition, 6 of 9 participants saw clinically relevant improvements in overall AHS scores with THS.

The findings of this study are promising but should be considered exploratory in nature due to the small sample size. It was designed as a pilot trial with the intent to assess any significant trends and determine the sample size needed for future clinical trials. In addition, THS's overall impact may have been truncated by the three extra shots taken in the active arm. One participant from each treatment arm vomited once within 60 minutes of alcohol administration, and the implication of this on the findings is unknown. No other adverse effects were reported. While the study controlled for food and water consumption, the effect of marginal differences could not be accounted for.

THS showed positive signals in the prevention of alcohol‐induced hangover, especially headaches. However, larger studies with appropriate sample sizes are needed.

## CONFLICT OF INTEREST

The authors declare no conflicts of interest.

## AUTHOR CONTRIBUTIONS

Conceptualization: Der Yu Wang, Sheel Patel, Kimberly Maiton, Kate M. O'Dell, Nancy N. Nguyen, Sachin A. Shah.

Formal Analysis: Der Yu Wang, Sheel Patel, Kevin Pham, Sachin A. Shah.

Methodology: Der Yu Wang, Sheel Patel, Kimberly Maiton, Kate M. O'Dell, Nancy N. Nguyen, Sachin A. Shah.

Resources: Der Yu Wang, Sheel Patel, Kate M. O'Dell, Nancy N. Nguyen, Sachin A. Shah.

Writing – Original Draft Preparation: Der Yu Wang, Sheel Patel, Sachin A. Shah.

Writing – Review & Editing: Der Yu Wang, Sheel Patel, Kevin Pham, Kate M. O'Dell, Nancy N. Nguyen, Sachin A. Shah.

All authors have read and approved the final version of the manuscript.

Sachin A. Shah had full access to all of the data in this study and takes complete responsibility for the integrity of the data and the accuracy of the data analysis.

## TRANSPARENCY STATEMENT

Sachin Shah affirms that this manuscript is an honest, accurate, and transparent account of the study being reported; that no important aspects of the study have been omitted; and that any discrepancies from the study as planned (and, if relevant, registered) have been explained.

## ETHICAL STATEMENT

Approved by the Institutional Review Board at the University of the Pacific, Stockton, California.

## DISCLAIMER

The views expressed in this material are those of the authors and do not reflect the official policy or position of the U.S. Government, the Department of Defense, the Department of the Air Force, or the University of the Pacific.

## Data Availability

The data that support the findings of this study are available on request from the corresponding author. The data are not publicly available due to privacy restrictions.
